# Complications and consequences: short-term harm has long-term impact

**DOI:** 10.1016/j.bjao.2023.100233

**Published:** 2023-10-19

**Authors:** Alexander I.R. Jackson, S. Ramani Moonesinghe, Michael P.W. Grocott

**Affiliations:** 1 Perioperative and Critical Care Theme, NIHR Southampton Biomedical Research Centre, University Hospital Southampton NHS Foundation Trust, Southampton, UK; 2 Integrative Physiology and Critical Illness Group, Clinical and Experimental Sciences, Faculty of Medicine, University of Southampton, Southampton, UK; 3 Centre for Peri-Operative Medicine, Research Department for Targeted Intervention, University College London, London, UK; 4 University College London/University College London Hospitals National Institute Health Research Biomedical Research Centre, London, UK; 5 Department for Anaesthesia and Perioperative Medicine, University College London Hospitals, London, UK

**Keywords:** healthcare utilisation, long-term, morbidity, perioperative complications, postoperative complications, shared-decision making

## Abstract

In this editorial, we discuss a large observational study demonstrating increased healthcare usage and higher mortality over 2 yr in patients who experienced specific postoperative complications. These findings are in keeping with the existing literature and draw into focus the need for ongoing work to understand and communicate these long-term consequences to patients.

Complications after surgery are distressing and unpleasant for patients, a burden on health system resources, and have substantive long-term health implications. The study by Fowler and colleagues^1^ published in the September 2023 issue of *BJA Open* adds to the literature supporting the notion that complications are key determinants of long-term outcomes and health system burden after surgery.

The size of the study, with almost 50 000 patients, is a significant strength, as is the integration of primary and secondary care data, giving a more complete overview of long-term health than either in isolation. This must be balanced against the observational nature of the data, limiting the causal inference that can be made. It should also be noted that the study did not explore the totality of complications but rather identified a sentinel set of readily identifiable complications to explore their relationship with long-term outcomes.

However, in agreement with the existing literature, some conclusions can be drawn. Previous studies have consistently shown that postoperative complications are linked to increased short-term costs. Eappen and colleagues,^2^ working in the USA, demonstrated a two-to three-fold increase in short-term costs during the index admission associated with complications after major surgery. In Europe, Vonlanthen and colleagues^3^ showed an increase of up to five times in inpatient care cost in patients who experienced complications. The study by Fowler and colleagues^1^ suggests that this increased healthcare use extends beyond the index admission. Although all patients saw an increase in healthcare use after surgery, this rate was significantly higher in those who experienced the selected complications. The maximal increase was observed in the first 6 months; yet, a sustained effect was noted for the 2 yr duration of follow-up. Although the authors did not conduct a formal health economic analysis, there is a clear implication that there is a greater overall burden on resources over a prolonged period.

In addition, Fowler and colleagues^1^ identified a more than three-fold increase in the incidence of death within 2 yr of major surgery in patients who experienced a complication compared with patients who experienced an uncomplicated recovery. This observation is clearly limited by confounding in that patients identified as having a higher preoperative risk of adverse outcomes are more likely to experience both complications and death; a common causal relationship is present. In the absence of an adjustment for preoperative risk, this observation is difficult to interpret. However, several studies have adjusted for baseline risk, including the original study of Khuri and colleagues^4^ and the study by Moonesinghe and colleagues.^5^ In both cases, these studies demonstrated an increase in mortality, for up to 8 yr, after surgery in patients who experienced a complicated recovery, even when adjusted for baseline risk. Consistency of association in observational data is the best indicator of a true signal. Reassuringly, both studies^4^^,^^5^ used different methods of risk adjustment and outcome evaluation, yet arrived at the same conclusion: short-term complications after surgery are associated with long-term survival. Importantly, this counters any assertion that the observed variation in outcomes is driven simply by those at greater risk of experiencing more complications.

These findings raise important areas of consideration for both clinical practice and future research. First, it emphasises the complexity of the benefit–harm relationship surrounding surgery. Although short- and long-term mortality remain important, and valid outcomes should be reported,^6^ outcomes cannot be viewed through the binary lens of survival within a specified time period alone. Rather, clinicians must seek to understand the long-term implications of surgery and its possible complications for both patients and healthcare systems. The work by Fowler and colleagues^1^ builds on the existing literature, suggesting that even in survivors, there is ongoing increased healthcare use, which may be linked to reduced quality of life and patient experience and higher costs and resource utilisation.

Shared decision-making,^7^ also known as collaborative decision-making, where patients and clinicians discuss and decide on treatment options together, offers one possible means of ameliorating some of these effects. It may improve the patient experience and is associated with reduced surgical regret.^8^ The process may also result in those at highest risk of complications choosing not to undergo surgery, with consequent benefits to resource utilisation. Shared decision-making should not force this decision on high-risk patients, but rather it is rational that for patients who are at highest risk of complications or poor outcomes to weigh this against the relative benefit, and some may conclude that surgery is not their preferred choice.

Yet, this study also raises important questions regarding shared decision-making. We are fortunate to have tools that can predict short-term mortality with relative accuracy,^9^, ^10^, ^11^ alongside the widespread reporting of morbidity and mortality in cohort studies and clinical trials. However, our understanding of the long-term impact of surgery and its complications on patients' health and life experiences remains more limited. This means that, although we can discuss with relative accuracy the risk of death or a complication with a patient in a shared decision-making discussion, it is much harder to talk with any certainty about how their overall health, quality of life, or function may be in the months and years after surgery.

Future research should build on the work of Fowler and colleagues^1^ to develop a more complete understanding of postoperative recovery. This is a complex and dynamic process, which may not be best evaluated by point estimates.^12^ Instead, longitudinal research with multiple time points should aim to map recovery trajectories, with a focus on the outcomes that are important to patients.

Ideally, this should be paired with further work to evaluate shared decision-making. Shared decision-making can take many forms and be conducted in different ways.^13^ Future studies should try to understand the effect of these differing approaches and identify the groups in which each may be most effective. It is of particular importance in such work to focus on the views and experiences of patients.

Fowler and colleagues^1^ have highlighted important issues. Postoperative recovery is about more than the index admission. Instead, we should consider the long-term consequences of surgery—both favourable and unfavourable. With increasing consistency, short-term harm appears to have longer term consequences. We are beholden to seek to understand these consequences and how best to explain them to our patients. If this can be done, it will be of benefit not only to them but also to healthcare systems and wider society as well.

## Author’s contributions

AIRJ: First draft, revision.

MPWG: Conception, comment and revision.

SRM: Comment and revision.
